# *ABI3 *ectopic expression reduces *in vitro *and *in vivo *cell growth properties while inducing senescence

**DOI:** 10.1186/1471-2407-11-11

**Published:** 2011-01-11

**Authors:** Flavia RM Latini, Jefferson P Hemerly, Beatriz CG Freitas, Gisele Oler, Gregory J Riggins, Janete M Cerutti

**Affiliations:** 1 Genetic Bases of Thyroid Tumors Laboratory, Division of Genetics and Division of Endocrinology, Universidade Federal de São Paulo, SP, Brazil; 2 Department of Neurosurgery, Johns Hopkins University School of Medicine, Baltimore, MD, USA

## Abstract

**Background:**

Mounting evidence has indicated that *ABI3 *(ABI family member 3) function as a tumor suppressor gene, although the molecular mechanism by which ABI3 acts remains largely unknown.

**Methods:**

The present study investigated *ABI3 *expression in a large panel of benign and malignant thyroid tumors and explored a correlation between the expression of ABI3 and its potential partner ABI3-binding protein (ABI3BP). We next explored the biological effects of *ABI3 *ectopic expression in thyroid and colon carcinoma cell lines, in which its expression was reduced or absent.

**Results:**

We not only observed that *ABI3 *expression is reduced or lost in most carcinomas but also that there is a positive correlation between *ABI3 *and *ABI3BP *expression. Ectopic expression of *ABI3 *was sufficient to lead to a lower transforming activity, reduced tumor *in vitro *growth properties, suppressed *in vitro *anchorage-independent growth and *in vivo *tumor formation while, cellular senescence increased. These responses were accompanied by the up-regulation of the cell cycle inhibitor *p21 *^WAF1 ^and reduced ERK phosphorylation and *E2F1 *expression.

**Conclusions:**

Our result links *ABI3 *to the pathogenesis and progression of some cancers and suggests that ABI3 or its pathway might have interest as therapeutic target. These results also suggest that the pathways through which *ABI3 *works should be further characterized.

## Background

The ABL-Interactors (ABI) proteins were initially identified as binding partners of c-ABL tyrosine kinase, a non-receptor tyrosine kinase whose activation results in cell growth, cell transformation and cytoskeletal reorganization. It has been suggested that the ABI1 (ABI family member 1) and ABI2 (ABI family member 2) act as tumor suppressor genes [1,2].

ABI3 (ABI family member 3) is the third member of ABI protein family that, similar to ABI1 and ABI2, is involved in membrane ruffling and lamellipodia formation, which suggest the involvement of ABI3 in cell motility [3,4].

It has been shown that *ABI3 *expression is lost in invasive cancer cell lines, despite its ubiquitous expression in normal tissues [4]. In addition, ectopic expression of *ABI3 *in metastatic cell lines caused a marked reduction in cell motility and exhibited significant reduction in tumor metastatic potential *in vivo *[3]. Moreover, over-expression of *ABI3 *potently blocked PDGF-stimulated membrane ruffling in mammalian cells [5]. Although these reports indicate that *ABI3 *loss may play a role in the pathogenesis and/or progression of certain cancers, the precise function of ABI3 in human cancer and the potential signaling pathway and downstream effectors of ABI3 remain unclear.

A yeast two-hybrid system with the SH3 domain of ABI3 as the bait protein was used in order to identify novel components of ABI3 signaling pathways. ABI3BP (ABI3Binding Protein) was originally identified as an SH3 domain-binding molecule of ABI3 [4].

We previously described that *ABI3BP *expression is reduced in malignant thyroid samples, compared to normal thyroid and benign lesions [6-8]. Furthermore, we demonstrated that ectopic expression of *ABI3BP *decreased tumor growth properties *in vitro *and *in vivo*, while induced senescence [8]. Other studies have shown that *ABI3BP *was also associated with pathogenesis of lung cancers by virtue of its reduced expression in all lung cell lines and lung primary tumors [9]. The authors also demonstrated that *ABI3BP *is potentially associated with pathogenesis of colon, ovary and thyroid, as its expression was reduced in primary tumors compared to paired normal samples [9].

Our hypothesis is that, similar to *ABI3BP*, *ABI3 *expression might be reduced in thyroid carcinomas and possibly plays a functional role in the pathogenesis and/or progression of thyroid tumors as well as other cancers.

To test this hypothesis, we investigated the expression of *ABI3 *in thyroid benign and malignant lesions. We found a decreased expression of *ABI3 *in thyroid carcinomas. We next explored the biological role of *ABI3 *in thyroid and colon carcinoma cells. We showed that *ABI3 *suppressed the *in vitro *and *in vivo *transformation, induced senescence and inhibited the oncogenic signaling. These findings demonstrate the tumor suppressing activity of ABI3 and suggest that it may be a target for therapy.

## Methods

### Tissue samples

A total of 81 thyroid tissue specimens obtained from patients undergoing thyroid surgery for thyroid disease at Hospital São Paulo, Federal University of São Paulo, Brazil, were used for this study. Samples were frozen immediately after surgical biopsy and stored at -80°C. The samples included 7 normal thyroid tissues, 21 follicular thyroid adenomas, 14 Hürthle cell adenomas, 15 follicular thyroid carcinomas, 6 Hürthle cell carcinomas and 18 papillary thyroid carcinomas. All tissue samples were obtained with informed consent according to established Human Studies Protocols at Federal University of São Paulo. The study of patient materials was conducted according to the principles expressed in the Declaration of Helsinki.

### RNA extraction, cDNA synthesis and quantitative PCR (qPCR)

To investigate the level of *ABI3 *expression in thyroid tumors, total RNA and cDNA synthesis was performed as previously described [10]. An aliquot of cDNA was used in 20 μl PCR reactions containing TaqMan universal PCR master mix, 10 μM of each specific primer and FAM-labeled probes for the target gene (*ABI3*) and VIC-labeled probe as the reference gene (*S8*) (TaqMan^®^Gene Assays on Demand; Applied Biosystems, Foster City, CA). Gene expression was normalized to the average of *S8 *expression and relative expression was calculated as described earlier [11,12].

### Correlation of *ABI3 *and *ABI3BP *expression in thyroid tumors

The level of *ABI3 *expression was correlated with the level of *ABI3BP*, which was previously investigated in this set of samples [8].

### Cell Culture

A follicular thyroid carcinoma cell line (WRO) and a colon cancer-derived HT-29 cell line (ARO) [13] were grown in DMEM (Invitrogen Corp., Carlsbad, CA) supplemented with 10% FBS (Invitrogen Corp.), 100 units/mL of penicillin and 100 μg/mL streptomycin in a humidified incubator containing 5% CO_2 _at 37°C [14,15].

### Generation of stable tranfected clones Expressing of ABI3

Plasmid encoding the full-length cDNA of human *ABI3 *was kindly donated by Dr. Satoru Matsuda (Nagoya University School of Medicine, Nagoya, Japan). To establish cell lines expressing *ABI3*, 10 μg of DNA construct were transfected into WRO and ARO cells by electroporation using a Gene Pulser II (Bio-Rad Laboratories Inc., Hercules, CA). ARO and WRO cells transfected with pcDNA3.1 vector were used as the negative controls. Clones were isolated after 3 weeks of selection with G418 (800 μg/mL). At least six G418-resistant clones from each transfection were isolated, expanded, maintained on G418 (400 μg/mL) and tested for *ABI3 *expression by qPCR. To this end, total RNA extracted from each clone was used for cDNA synthesis as described [8]. An aliquot of cDNA was used in a 20 μL PCR reaction containing SYBR Green PCR Master Mix (Applied Biosystems) and 200 nM of each primer for target or reference genes. qPCR was performed in triplicates and the threshold cycle (Ct) was averaged (SD ≤1). Primer sequences for *ABI3 *and *S8 *(internal control) were as follows: *ABI3 *sense 5'-CAGGTGGAAGCCCGTGTAAG-3' and antisense 5`-AGTGGCTAAGGTGCCGATCTC-3', yielding a product of 89 bp; *S8 *sense 5'-TGAAAGGAAAAAGAATGCCAAAA-3' and antisense 5'-CACTGTCCCGGCCTTGAA-3', yielding a product of 96 bp. Gene expression was normalized to the average of *S8 *and relative expression was calculated as described [11,12]. For each cell line, two independently isolated clones that expressed *ABI3 *at similar levels and two pcDNA3.1 clones were used for further *in vitro *and *in vivo *experiments.

### Transformation assay

About 5 × 10^6 ^WRO cells were transfected with 10 μg of the *ABI3 *DNA construct as described above. Control plates were transfected with pcDNA3.1. After 3 weeks of selection with G418 (800 μg/mL), cells were fixed in 10% acetic acid and 10% of methanol and stained with 1% crystal violet. G418-selected colonies were counted. Each experiment was performed in triplicate.

### Proliferation Assay

Stably transfected clones for ARO and WRO were analyzed with the 3-(4,5-dimethylthiazol-2-yl)-2,5-diphenyl-tetrazolium bromide (MTT) assay as described [8,16]. In brief, 2 × 10^4 ^cells were seeded in 35-mm plates on day 0. Cell growth was measured from day 1 to 5 by adding 0.5 mg/mL of MTT (Sigma-Aldrich, St. Louis, MO) to the medium at 37°C for 3 hours. The medium was removed and purple formazan crystals were dissolved by adding acid isopropanol. The absorbance of the supernatant was measured at 560 nm.

### Quantification of apoptotic cells by annexin-V labeling

To test whether ectopic expression of *ABI3 *induces apoptosis, 2 × 10^4 ^cells were seeded in 35-mm plates and double-stained with Annexin V and Nexin 7-AAD according to the manufacturer's recommendations (Guava Nexin method; Guava Technologies). Cell-associated fluorescence was analyzed by the Guava PCA flow cytometer (Guava Technologies). Results are expressed as the percentage of apoptotic positive cells. Both early apoptotic (annexin V-positive) and late apoptotic (annexin V- and 7 AAD-positive) cells were included in the analysis. Experiments were performed in quintuplicates.

### Cell viability assay

ARO Cells (2 × 10^4^) were seeded in 35-mm plates. Cells were mixed with Guava ViaCount Reagent and allowed to stain for 10 minutes (Guava Technologies, Hayward, CA). Viable cells were quantified using a Guava Personal Analyzer (PCA) flow cytometer (Guava Technologies) following the manufacturer's specifications. Experiments were performed in quintuplicates.

### Cell cycle analysis

ARO cells (2 × 10^5^) were seeded in 35-mm dishes. After synchronization of the cells by serum starvation for 24 hours, cells were replaced with DMEM medium supplemented with 10% FBS for 24 hours. Cells were fixed in 70% ethanol for 1 hour, labeled with Guava Cell Cycle Assay reagent and analyzed using Guava PCA flow cytometer (Guava Technologies), according to manufacturer's recommendations. Experiments were performed in quintuplicates.

### Expression of p21^WAF1 ^and E2F1 by qPCR

The transcript levels of *p21*^*WAF1 *^and *E2F1 *were tested in stably expressing *ABI3 *ARO and WRO cells and controls, as described [8].

### Western blot analysis

Western blot analysis was performed as described [8]. Briefly, membranes were blocked and incubated overnight at 4°C with anti-phospho-ERK (pERK; dilution 1:1000), anti-phospho-AKT (pAKT; dilution 1:400) and anti-α- Tubulin (dilution 1:1000). Detection was carried out using the SuperSignal West Pico chemiluminescent substrate (Pierce, Rockford, IL, USA).

### Cellular senescence

Senescence-associated (SA) β-gal staining was performed as described [17]. Briefly, ARO and WRO cells (2 × 10^4^) were seeded in 35-mm plates. Cells were washed twice with PBS, fixed for 15 minutes and stained with 1 mg/mL 5-bromo-4-chloro-3-inolyl-b-D-galactoside (X-gal) in buffer (dimethyformamide, 40 mM citric acid/sodium phosphate pH 6.0, 5 mM potassium ferrocyanide, 5 mM potassium ferricyanide, 150 mM NaCl and 2 mM MgCl_2_). Cells were incubated at 37°C in 5% CO_2 _for 18 hours and washed twice with PBS. Cells were examined using a light microscope and counted in 5 optical fields (100×). Data represents mean of an experiment performed in quintuplicates.

### Matrigel invasion assay

Cell invasion was analyzed using BioCoat Matrigel Invasion Chamber according to the manufacturer's recommendation (Becton Dickinson, Bedford, MA). WRO cell clones were added to the invasion or control chambers at a density of 2.5 × 10^4 ^and, after 24 hours, cells remaining above the insert membrane were removed by gentle scraping with a sterile cotton swab. FBS was used as chemoattractant. Cells that had invaded through the Matrigel to the bottom of the insert were fixed and stained with rapid panoptic LB (Laborclin, Brazil) and mounted. Cells were examined using a light microscope and counted in 3 optical fields (100×). Experimental and control groups were performed in triplicates. The percentage of invasion cells was determined by the mean of cells invading through Matrigel insert membrane divided by the mean of cells migrating through control insert membrane X100.

### Cell Migration from spheroids

Because high migration capacity might be correlated with cell spreading and metastasis *in vivo*, migration from spheroids was assayed as previously described [8]. Briefly, spheroids were prepared by seeding WRO cells in DMEM supplemented with 10% FBS, onto 35-mm tissue culture dishes coated with 0.75% Noble agar. Cells were cultured until spheroids were formed and single spheroids were placed at the center of each well of a 24-well plate. At least 12 single spheroids from each selected clone were cultured. The area covered by cells spreading out from the spheroid was measured every 24 hours for a period of 6 days. The areas of spheroids were calculated as described [8].

### Anchorage-independent growth

Anchorage-independent growth was assessed by a double-layer soft agar assay. Initially, 60-mm dishes were layered with 0.5% agar and 1× complete medium. Next, ARO cells (1.5 × 10^4^) were suspended in 1× complete medium and 0.35% agar and seeded in triplicate over a bottom layer of solidified agar. The dishes were incubated at 37°C in 5% CO_2_. After 3 weeks, colonies greater than 20 μm in diameter were counted. Colony formation rate was calculated as percentage of total seeded cells. Two independent experiments were performed.

### Nude mouse xenograft model

Four to five week old male athymic nude (*nu*/*nu*) mice were maintained according to the guidelines of the Division of Animal Resources at the Federal University of São Paulo. ARO stable cell clones were suspended in sterile PBS to 2 × 10^6^/200 μL and injected subcutaneously into the flank of mice. Mice were then monitored biweekly during three weeks. Tumor volume was calculated by the rotational ellipsoid formula: V = A × B^2^/2 (A = axial diameter; B = rotational diameter). Tumor tissues were collected and embedded in paraffin for conventional histology or were stored at -80°C.

### Statistical analysis

The relative expression values were log transformed before the application of statistical analysis. Pearson correlation coefficient was used to verify the correlation between *ABI3 *and *ABI3BP *expression. *In vitro *results were log transformed and analyzed by a Student's *t *test. *In vivo *results were analyzed by the Wilcoxon test. Significance is presented as p value of <0.05 (*), < 0.01 (**) and < 0.001 (***).

## Results

### *ABI3 *expression is reduced in malignant thyroid lesions

**Figure 1:**
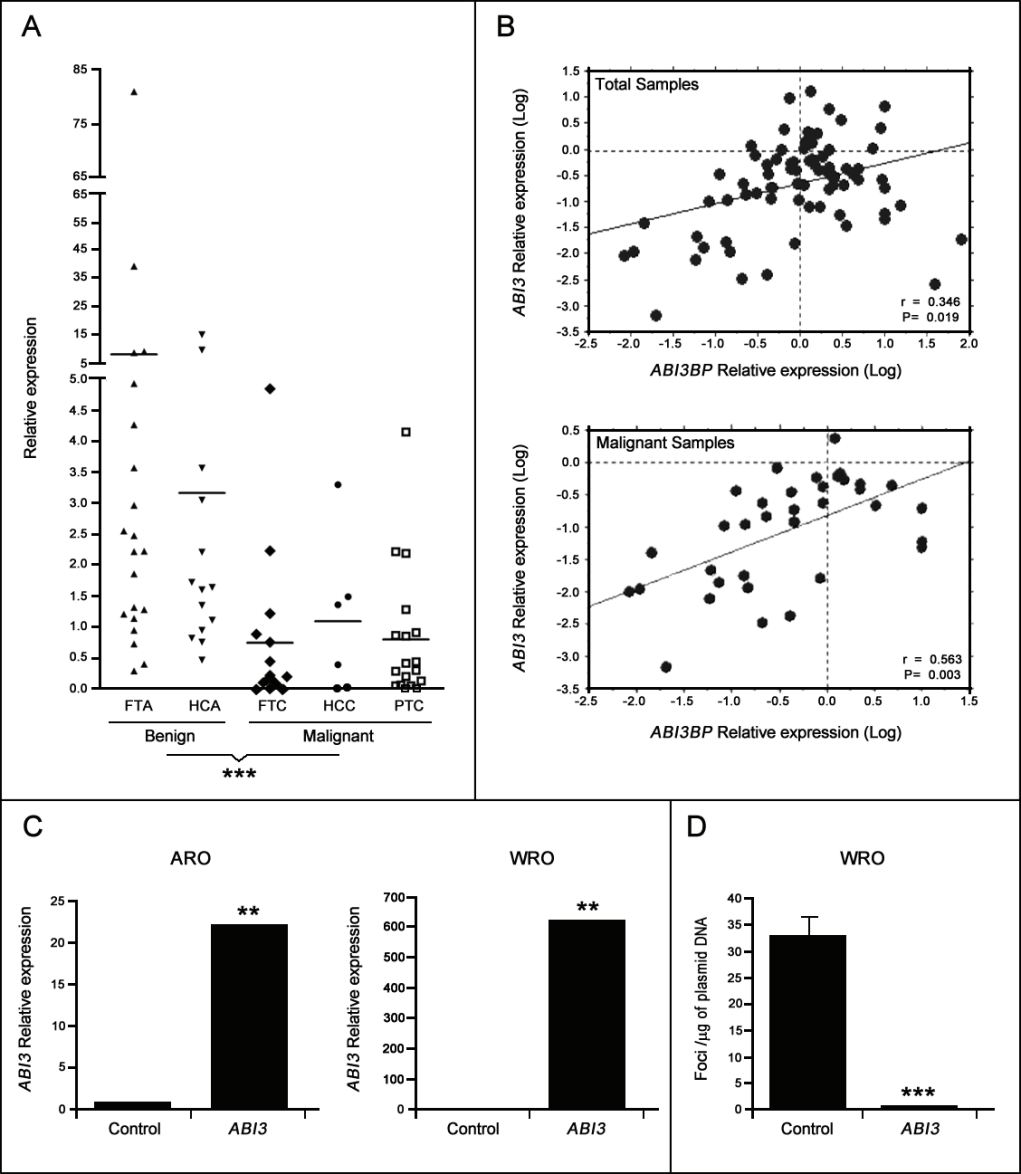
**Status of *ABI3 *expression in thyroid tumors and in carcinomas cell lines**. (A) *ABI3 *expression is reduced in most thyroid malignant tumors (p < 0.001). (B) A positive correlation was observed between levels of *ABI3 *and *ABI3BP *expression in the panel of thyroid samples, mainly in malignant tumors. (C) *ABI3 *expression in control cells (cells transfected with empty vector) or clones expressing *ABI3 *(two of each cell line). (D) *ABI3 *ectopic expression reduced focus formation. The data represents the mean ± SD of the experiment performed in triplicates. FTA, Follicular thyroid adenomas; HCA, Hürthle cell adenomas; FTC, follicular thyroid carcinomas; HCC, Hürthle cell carcinomas; PTC, papillary thyroid carcinomas. *p *<*0.05, **p < 0.01, ***p≤ 0.001.

To test the possibility that *ABI3 *expression is associated with thyroid tumor malignancy, we examined mRNA expression in a panel of thyroid tumors specimens and normal thyroid. As demonstrated by qPCR, *ABI3 *expression was reduced in a high percentage of thyroid carcinomas while it was expressed in most of benign lesions and normal thyroid (p≤0.001; Figure 1A). Since we previously investigated the level of *ABI3BP *in the aforementioned set of samples [8], we next correlated the expression of *ABI3 *with the expression observed for *ABI3BP*. We found a medium positive correlation between reduced expression of *ABI3 *and *ABI3BP *(*r *= 0.346; p = 0.019; Figure 1B). However, a large positive correlation was observed in malignant lesions (*r *= 0.564; p = 0.003; Figure 1B). These findings and those reported from two-hybrid system suggest that *ABI3 *and *ABI3BP *may act through a common signaling pathway, although biological evidences are still needed to demonstrate this hypothesis.

### Ectopic expression of *ABI3 *in human carcinoma cell lines

To investigate a functional role of *ABI3 *in cancer development, *ABI3 *expression was tested in a panel of cell lines derived from human cancers. A thyroid follicular cell line (WRO) and a colon cancer cell line (ARO), which did not express or expressed at very low levels, were chosen (Figure 1C). A construct expressing *ABI3 *and an empty vector (control) were transfected into ARO and WRO cell lines. Seven transfected clones from each cell line were subsequently tested by qPCR. Two clones with similar level of *ABI3 *expression and two clones from control group were chosen for *in vitro *and *in vivo *studies (p < 0.01; Figure 1C).

### Expression of *ABI3 *suppresses focus formation

To determine the effects of the *ABI3 *on transformation of human cancer cells we first determined the effects of *ABI3 *on cells growth in a focus formation assay. WRO cells were stably transfected with vector expressing *ABI3*. Control transfections were also performed with empty vetor. G418-selected colonies were counted after two weeks. WRO cells transfected with empty vector formed numerous foci (32.70 foci/μg of plasmid DNA). In contrast, ectopic expression of ABI3 reduced the number of colonies formed (0.63 foci/μg of plasmid DNA) (p < 0.001; Figure 1D). This finding suggests that *ABI3 *strong inhibited foci formation.

### The ectopic expression of *ABI3 *reduces cell proliferation

**Figure 2:**
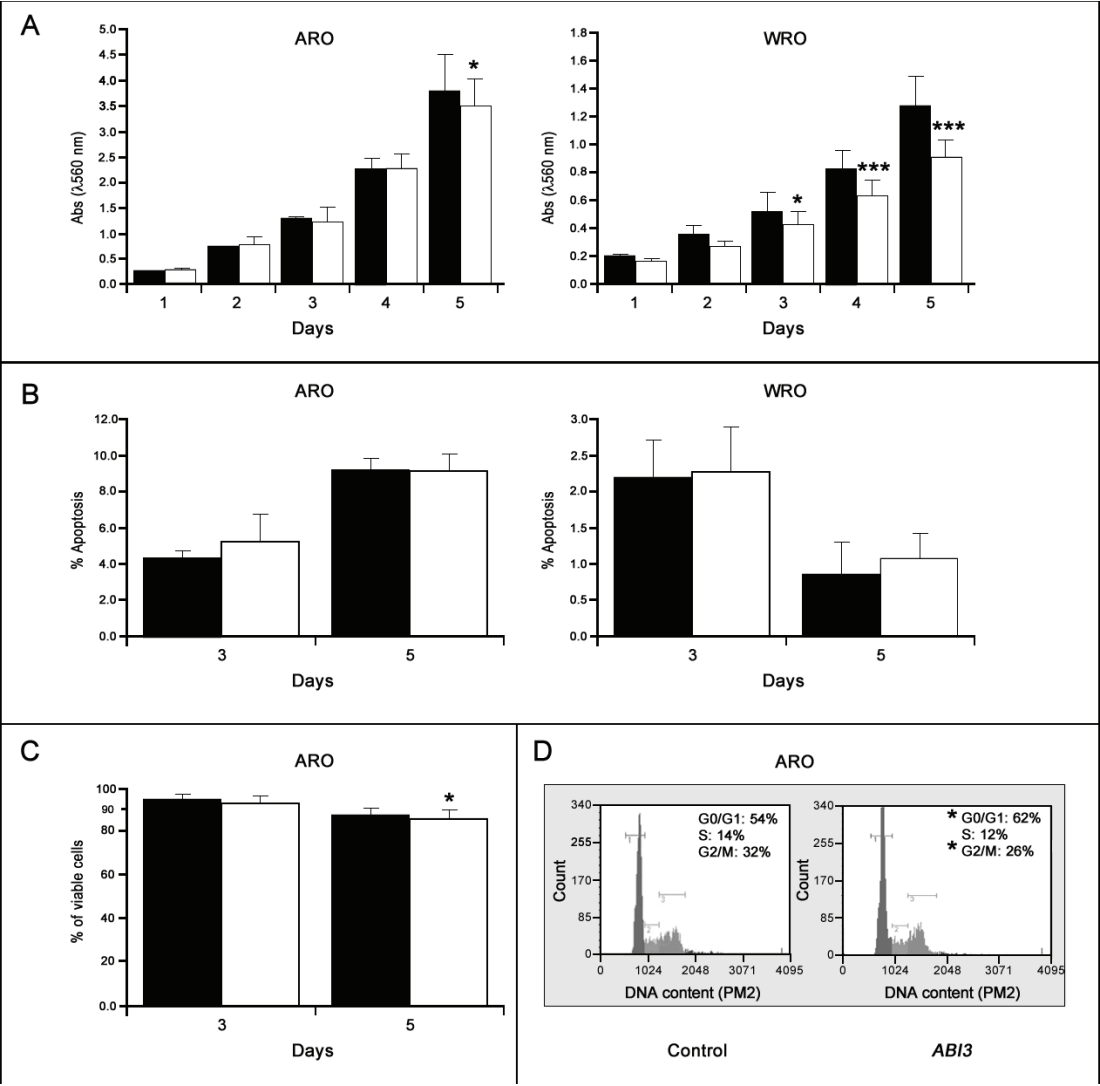
***ABI3 *ectopic expression reduced cell proliferation, cell viability and arrested cells in G0/G1 phase**. (A) Growth of cells is suppressed following *ABI3 *expression, mainly in WRO cell line. (B) The percentage of apoptotic cells was not significantly altered in cell lines expressing *ABI3*, compared to control cells. (C) *ABI3 *expression reduced cell viability in ARO cells, mainly at day 5 where a more evident effect was observed in the proliferation assay. (D) *ABI3 *expression increased the percentage of cells in G_0_-G_1 _at the expense of G_2_-M phase. Black bars correspond to control clones (*n *= 2) and white bars correspond to *ABI3 *expressing clones (*n *= 2). *p < 0.05 and *** p < 0.001.

To confirm the effects of the ABI3 on malignant transformation, we next examined the effects of ectopic expression of *ABI3 *in growth rate. *ABI3 *induced a growth inhibitory effect in the two cell lines as assessed by MTT assays, mainly at day 5 (Figure 2A). The data represents the mean ± SD of two experiments performed in triplicates.

### *ABI3 *expression increases the percentage of cells in G0/G1 phase and reduces cell viability but did not induce apoptosis on carcinoma cells

The ability of *ABI3 *to inhibit cell growth could be due to the cell cycle arrest and/or apoptosis. Therefore we investigated the effect of *ABI3 *on apoptosis. Although we observed a trend toward increased apoptosis in WRO cells expressing *ABI3 *when compared to control cells, the growth attenuation was, however, not accompanied by a corresponding increase in apoptotic cells (Figure 2B). The ABI3-induced growth suppression in was further studied by flow cytometric analysis, which revealed a decrease in cell viability (Figure 2C) and cell cycle arrest at the G0/G_1 _phase (p < 0.05, Figure 2D).

### *ABI3 *induces the expression of p21^WAF1 ^and reduces E2F1 expression and phosphorylation of ERK

**Figure 3:**
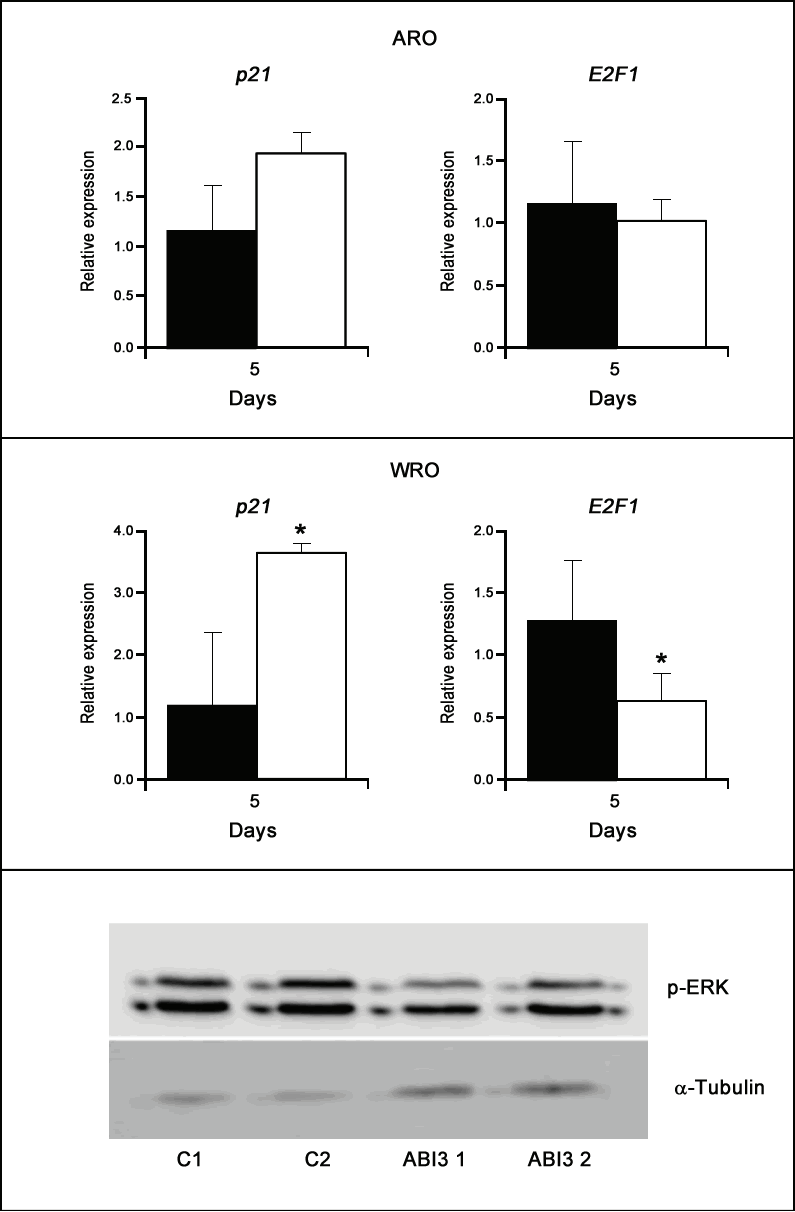
***ABI3 *induced p21**^**WAF1 **^**expression while reducing ERK phosphorylation and *E2F1 *expression in carcinoma cell lines**. (**A**. two first panels - two upper panels) An increase in *p21*^*WAF1 *^and decrease in *E2F1 *expression was observed following *ABI3 *ectopic expression. Black bars correspond to control cells and white bars correspond to *ABI3 *expressing cells. Graphs show mean ± SD of two clones for each transfectants. (**B.** third panel-bottom) ABI3 ectopic expression decreases phosphorylated ERK (p-ERK) in WRO cells. Results from two selected clones are show. C1 and C2 (controls clones) and ABI3 1 and ABI3 *2 *(clones expressing *ABI3*). α-Tubulin was used as internal control. *p < 0.05.

Since *p21*^*WAF1 *^inhibit and *E2F1 *promotes cell cycle progression, we tested the transcripts levels of expression of *p21*^*WAF1 *^and *E2F1 *following *ABI3 *expression by quantitative PCR. Stable expression of *ABI3 *in WRO cells induced *p21*^*WAF1 *^while reduced *E2F1 *expression at days 3 and 5 post-seeding (p < 0.05; Figure 3A).

Although at lower levels, the expression of *p21*^*WAF1 *^was induced and *E2F1 *was reduced in ARO cells expressing *ABI3*. We next tested phosphorylation of ERK, which is known to play a pivotal role in cell proliferation. We observed a decrease of ERK phosphorylation in *ABI3*-expressing WRO cells compared to control cells. Two clones from each group are shown independently (Figure 3B). No effect was observed in AKT phosphorylation (data not shown).

### *ABI3 *induces senescence in carcinoma cells

**Figure 4:**
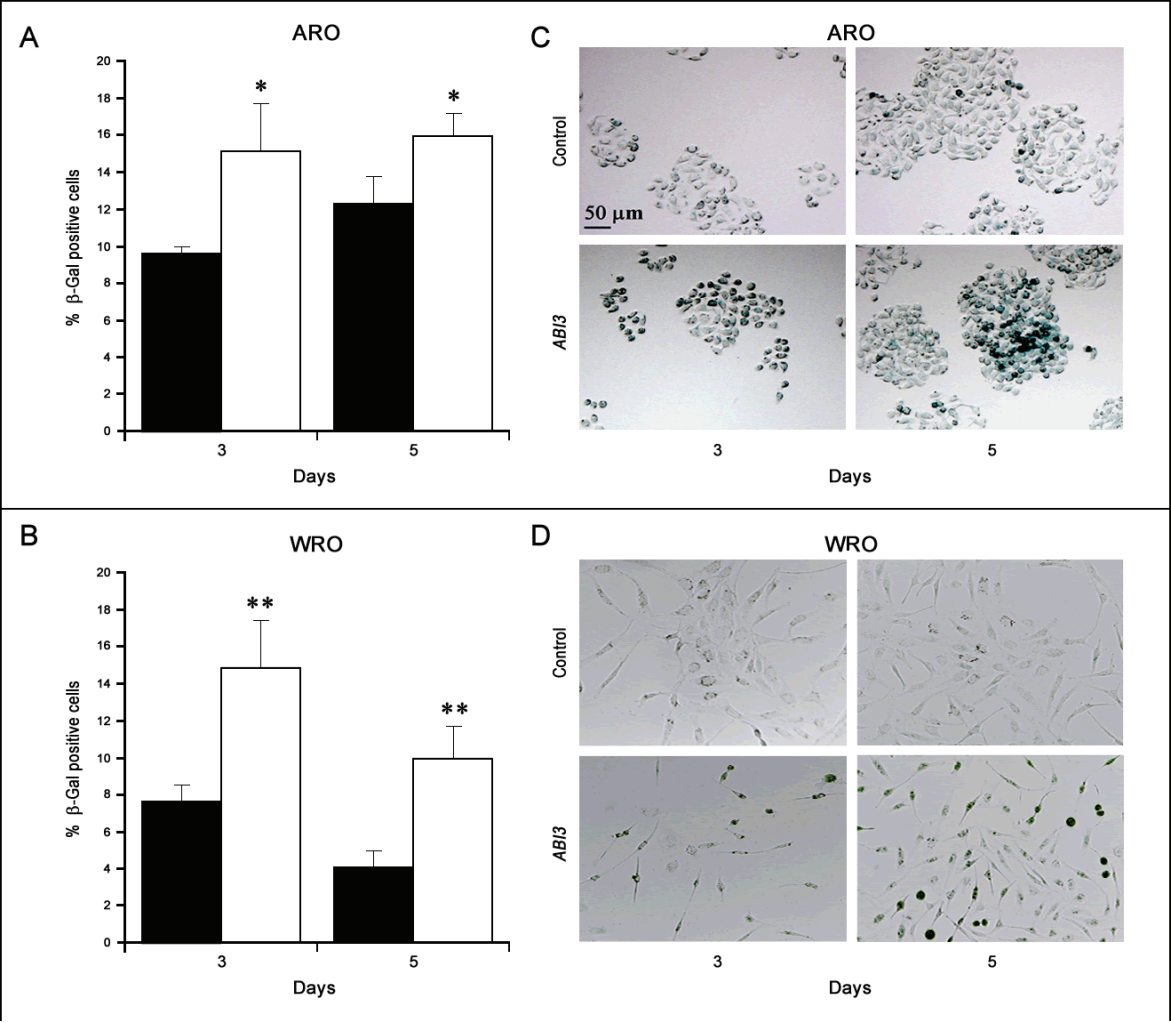
***ABI3 *ectopic expression induced senescence in carcinoma cell lines**. The percentage of β-galactosidase positive cells was higher in ARO (A) and WRO (B) cells expressing *ABI3 *than in control cells. Representative results illustrate increased senescence-associated β-galactosidase (SA-β-gal) staining in ARO (C) and WRO (D) cells following *ABI3 *expression on days 3 and 5. *p < 0.05 and **p < 0.01.

The number of β-Gal positive cells was higher in ARO and WRO cells expressing *ABI3 *at days 3 and 5 post-seeding (Figure 4A and 4B). Representative results are shown on Figure 4C and 4D. As predicted from cell proliferation and cell cycle assays, the effect was higher in WRO cells (p < 0.01) than in ARO cells (p < 0.05).

### *ABI3 *expression effects on cells migration

**Figure 5:**
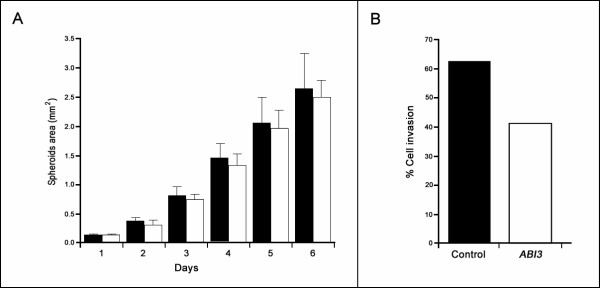
**Effect of *ABI3 *expression in invasion and migration**. (A) WRO cells expressing *ABI3 *had a lower ability to migrate. The graphs display the mean ± SD of at least 12 spheroids of each clone. (B) The percentage of invasive cells through the Matrigel assay was lower in WRO cells expressing *ABI3*. Black bars correspond to control clones (*n *= 2) and white bars correspond to *ABI3 *expressing clones (*n *= 2).

Given that *ABI3 *was previously associated with a marked reduction in cell motility, we here tested using spheroid assay. Expression of *ABI3 *reduced cell migration and invasion of WRO cells (Figure 5A and 5B), although it was not considered statistically significant. ARO cells did not form spheroids.

### *ABI3 *reduces tumor growth in nude mice

**Figure 6:**
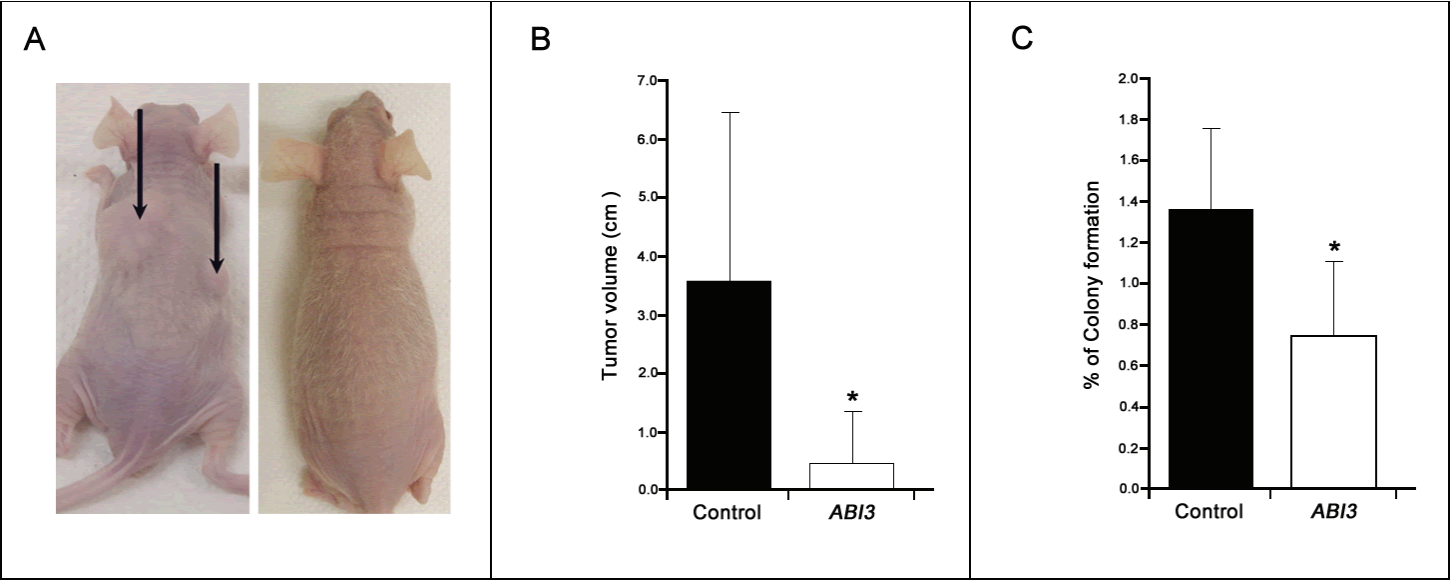
***ABI3 *reduced tumor formation in nude mice and the ability to growth in semi-solid medium**. (A) Representative animals that received either control cells (left panel) or cells expressing *ABI3 *(right panel) are shown. The arrows pointed out the formed tumors. (B) Tumorigenicity experiments are summarized in the graph showing mean ± SD of the volume of tumors after inoculation of ARO cells. (C) The percentage of colony formation in soft agar is significantly decreased in ARO cells expressing *ABI3 *in comparison with control cells. Experiments were performed in triplicates. *p < 0.05.

ARO cell line was selected for *in vivo *assay based on the fact that ARO cells form large tumors in *nude *mice [18], while WRO form smaller tumors with a long latent period. ARO cells expressing *ABI3 *did not form tumors in nude mice (*n *= 3) or formed a very small tumor (0.65 ± 1.17 cm^3^; *n *= 5). In contrast, control mice had extensive tumors (3.62 ± 2.84 cm^3^; *n *= 8; Figure 6A). The results are graphically represented as mean of tumor volume (p = 0.027; Figure 6B). Tumors were processed for routine histology and immunohistochemical analysis. H&E staining revealed no histological differences among the tumors. Neither lung nor lymph node metastases were found in any mice.

### *ABI3 *affects anchorage-independent cell growth

Since WRO cells had a very low colony-forming efficiency in agar, ARO cells were selected for its ability to growth in soft agar [18]. Following *ABI3 *expression, a significant reduction of the ability of the cells to grow in semi-solid media was observed (p < 0.05; Figure 6C).

## Discussion

We have previously shown that *ABI3BP *expression is lost in most malignant carcinomas. We also provided evidence that ectopic expression of *ABI3BP *in *ABI3BP*-deficient carcinoma cell lines inhibited *in vitro *and *in vivo *cell growth [8]. Based on our previous findings [6,8] and on the fact that ABI3BP was originally described as an ABI3-SH3-binding protein isolated by yeast two-hybrid technique [19], we investigated the expression of *ABI3 *in thyroid lesions.

In this study, we observed that *ABI3 *expression is lost or reduced in most malignant samples, compared to benign thyroid samples. Since both genes had similar patterns of expression, we subsequently investigated whether the expression of *ABI3 *and *ABI3BP *correlated in thyroid samples. We observed a positive correlation between *ABI3 *and *ABI3BP *expression, which was more evident in malignant lesions.

Our findings, in association with the fact that *ABI3 *expression is frequently lost in invasive cancer cell lines, and that *ABI3 *re-expression markedly inhibited cell motility and significantly reduced the formation of tumor metastasis *in vivo *[3], suggest that *ABI3 *loss of expression may play an important role in the pathogenesis and/or progression of several tumors subtypes.

Herein, we sought to investigate the consequences of stable expression of *ABI3 *on diverse steps of carcinogenesis including proliferation, transformation, survival, migration, and invasion *in vitro *and *in vivo*. To this end, we stably transfected *ABI3 *into a thyroid and a colon carcinoma cell lines [13].

We first demonstrated that *ABI3 *ectopic expression reduced cell transformation and suppressed proliferation of carcinoma cell lines, mainly in a follicular thyroid carcinoma cell line. The undetectable basal expression and high fold induction of *ABI3 *in the follicular thyroid carcinoma cell line could provide a potential explanation for the observed inhibitory effects of *ABI3 *on the cell proliferation. The different genetic background may also be responsible for the phenotypic differences.

Moreover, *ABI3 *expression was able to delay cell cycle progression and reduce cell viability at significant levels. Additionally, we found that *ABI3 *expression induces senescence, determined by positive results of SA-β-gal staining, which is a specific cellular senescence marker.

Even though it is not completely understood in detail how *ABI3 *promotes cell cycle arrest, reduces proliferation and induces senescence, our findings indicate that the *ABI3*-induced response was mediated by an increase in *p21*^*WAF1 *^expression, and down regulation of *E2F1 *expression.

Interestingly, p21^WAF1 ^is a major player in cell cycle control. Various mechanisms exist to regulate the levels of p21^WAF1^. Although tumor suppressor p53 is a major transcription factor involved in the regulation of p21^WAF1^, other factors including TGFβ, p73, SP1, Rac1, Rho, are known to induce the expression of p21^WAF1^. Therefore, the induction of p21^WAF1 ^expression could also occur in a p53-independent manner [20-22]. Once activated, p21^WAF1 ^exerts a negative effect on cell cycle progression by preventing the CDK2/cyclin E complex formation, leading to dephosphorylation (activation) of Rb and, thereby, preventing E2F-mediated transcriptional activation [21]. In addition to its role and cell cycle progression, previous studies provided evidences that p21^WAF1 ^can trigger senescence either in a p53-dependent and p53-independent manner [23].

In the present study, the effect of *ABI3 *expression on cell senescence, coupled with inhibition of cell cycle progression, up regulation of *p21*^WAF1 ^and down regulation of *E2F1 *expression, occurred in cancer cells in which p53 function is disrupted [24]. Therefore, in our model, p53 is unlikely to promote the ABI3-induced *p21*^*WAF1*^expression.

Although further analysis is needed to identify the underlying mechanism by which *ABI3 *induces *p21*^*WAF1 *^expression, our findings indicate that increase in *p21*^*WAF1 *^may mediate cell cycle arrest and senescence by blocking the CDk/Rb/E2F axis. It will be of interest to assess the effect of *ABI3 *expression on total Rb levels and its phosphorylation status.

In agreement with our findings, it was recently demonstrated that the apoptotic effect of iodine, in these cell lines, was mediated by mitochondrial pathway that involved p21^WAF1 ^accumulation in a p53-independent mechanism. The authors suggested that p21^WAF1 ^is believe to be an important molecule in drug induced tumor suppression, given that the block of p21^WAF1 ^significantly diminishes iodine-mediated apoptosis [25]. Up-regulation of p21^WAF1 ^has been reported to enhance apoptosis induced by antitumor agent in thyroid cancer cells in a p53-independent manner [26].

Although we did not observe changes in AKT phosphorylation when *ABI3 *was re-expressed, our findings corroborate with previous studies which demonstrated that *ABI3 *re-expression had no effect on AKT phosphorylation in v-Src transformed NIH3T3 and U87 MG cell lines [3].

In addition to increased growth rate, malignant transformation requires the acquisition of a number of tumor features. Although no significant differences were observed in migration and invasion assays, here we observed a direct correlation between *ABI3 *expression and anchorage-independent growth. Additionally, *ABI3 *significantly decreased xenograft growth in mice.

Interestingly, it has been previously demonstrated that *ABI3 *is a suppressive molecule in malignant cells [3]. The authors showed that *ABI3 *expression reduces cell motility and metastatic dissemination of a highly metastatic murine fibroblast transformed by v-Src (SRD) and the human glioblastoma cell line (U87 MG), while it did not interfere with cellular growth [3]. To examine molecular mechanism underlying *ABI3*-mediated effects in cell motility, the authors investigated whether the expression of Cdc42, Ras, Rac and Rho GTPases was affected by *ABI3 *expression. Neither significant activation, nor suppression was found in Cdc42, Rac, Ras and Rho. Interestingly, a marked reduction in phosphorylation of PAK2 was observed following expression of *ABI3*. Furthermore, the authors demonstrated that ABI3 and PAK2 colocalized at the leading edge of the cells [3].

These findings corroborate with our hypothesis that *ABI3 *loss could be common to other cancer types and suggest that, similar to other ABI-family members [1], *ABI3 *seems to function in a highly context-dependent way. Additional studies will be required to verify whether PAK2 and/or Rho and Rac small GTPases are affected by *ABI3 *expression in other cancer subtypes and to identify other mediator of cell motility.

In summary, our results indicate that *ABI3 *expression plays an important role in suppressing tumor growth and progression, given that its expression was significantly lower in malignant specimens compared to benign lesions and ectopic expression reduced the transforming phenotype of both cell lines. The identification of molecular events in the ABI3 pathway that control processes such as senescence, migration and invasion may suggest new therapeutic strategies for cancer.

## Conclusion

Our results indicate that *ABI3 *expression plays an important role in the pathogenesis and the progression of several cancers. A more detailed understanding of the pathway by which ABI3 contribute to senescence may lead to the development of novel agents that can suppress tumor development.

## Competing interests

The authors declare that they have no competing interests.

## Authors' contributions

FRL contributed to assay design, interpretation of the data, statistical analysis and drafted the manuscript. JHP performed *in vivo *assays, interpretation of the data and final art design. BF contributed to acquisition of the data, analysis and interpretation of the data. GO contributed to assay design and interpretation of the data. GJR participated in the design of the study and helped drafted and edited the manuscript. JMC directed the design and coordination of the study and contributed drafted the manuscript, responded to reviewers and interpreted the results. All the authors have read and approved the final version of the manuscript.

## Pre-publication history

The pre-publication history for this paper can be accessed here:

http://www.biomedcentral.com/1471-2407/11/11/prepub
